# An Occult HPV-Driven Oropharyngeal Squamous Cell Carcinoma Discovered Through a Saliva Test

**DOI:** 10.3389/fonc.2020.00408

**Published:** 2020-03-31

**Authors:** Kai Dun Tang, Sarju Vasani, Touraj Taheri, Laurence J. Walsh, Brett G. M. Hughes, Lizbeth Kenny, Chamindie Punyadeera

**Affiliations:** ^1^Saliva and Liquid Biopsy Translational Research Team, The School of Biomedical Sciences, Institute of Health and Biomedical Innovation, Queensland University of Technology, Brisbane, QLD, Australia; ^2^Institute of Health and Biomedical Innovation, Translational Research Institute, Brisbane, QLD, Australia; ^3^Department of Otolaryngology, Royal Brisbane and Women's Hospital, Brisbane, QLD, Australia; ^4^Department of Anatomical Pathology, Royal Brisbane and Women's Hospital, Brisbane, QLD, Australia; ^5^School of Medicine, The University of Queensland, Brisbane, QLD, Australia; ^6^The University of Queensland School of Dentistry, Brisbane, QLD, Australia; ^7^Department of Cancer Care Services, Royal Brisbane and Women's Hospital, Brisbane, QLD, Australia; ^8^Central Integrated Regional Cancer Service, Queensland Health, Brisbane, QLD, Australia

**Keywords:** human papillomavirus, oropharyngeal cancer, saliva, screening tools, biomarker

## Abstract

Oropharyngeal cancer (OPC) caused by human papillomavirus (HPV) is a rising global concern. Early lesions are small and are often located in difficult to access areas (such as the crypts of the tonsils or base of tongue). Unlike cervical cancer, there is no standard or routine screening program for HPV-driven OPC. HPV DNA from OPC tumors may shed directly into saliva, and this can be used as a biomarker for early diagnosis. In this study, we report the first-ever clinically occult OPC in an asymptomatic patient discovered through a saliva test. This case relied upon serial measurements of HPV-16 DNA in saliva, which fell to undetectable levels following low morbidity, curative treatment.

## Introduction

The incidence of high-risk human papillomavirus (HR-HPV−16,-18,-33) driven oropharyngeal cancer (OPC) is rapidly increasing in developed countries (1–3). HPV-driven OPCs have surpassed cervical cancer as the most common HPV-driven cancer in the USA. The prevalence of HR-HPV has been reported as 3.7% of the USA population, with a bimodal age distribution of incidence (4). It remains unclear why some individuals go on to develop OPC, while others clear the initial HPV infection (5). The strong association between HR-HPV infection and cervical cancer has led to screening programmes in primary healthcare settings, resulting in earlier diagnosis and a reduction in cancer deaths (6). Unlike cervical cancer, no screening test is available for OPC and current HPV vaccines have yet to demonstrate any reduction in future OPC development (7). Here, we report the first ever case of occult OPC detected as a direct result of a theoretical screening test—in this case HPV-16 DNA analysis in salivary oral rinse samples. Our clinical and pathological findings increase our understanding of both the natural history of the disease and the potential for wider screening to identify early stage OPC, facilitating less morbid treatments.

## Cases Presentation

An ongoing HPV-16 DNA prevalence study was approved by institutional ethics committees from the University of Queensland; Queensland University of Technology and the Royal Brisbane and Women's Hospital. A total of 665 cancer-free healthy individuals from Queensland Region, Australia between May 2016 and October 2017 were recruited. All participants gave written informed consent prior to sample collection.

**Figure 1:**
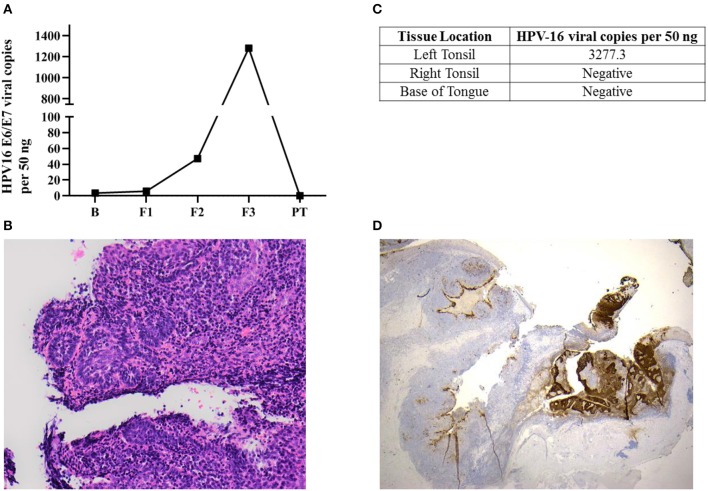
Occult oropharyngeal microcarcinoma detected based on a screening test through the serial measurements of salivary HPV-16 DNA. **(A)** HPV-16 DNA viral load in salivary oral rinse samples over time. B (Baseline); F1 (6 month follow-up); F2 (12 month follow-up); F3 (36 month follow-up); PT (2-week post-tonsillectomy). **(B)** Sections of the left tonsillar tissue found a 2 mm non-keratinising squamous cell carcinoma, with focal stromal invasion <1 mm, excised with clear margins. The remainder of the left tonsil showed follicular lymphoid hyperplasia. Hematoxylin-eosin (H&E x200) **(C)** HPV-16 DNA was only positive in left tonsillar tissue. **(D)** p16INK4a immunohistochemistry staining (IHC) x20: Diffuse positive brown staining for p16INK4a in tumor region comparing non-affected area in the left tonsil.

Six hundred and fifty cancer-free healthy individuals with sufficient amount of DNA were tested for oral HPV-16 DNA. Of these 3 have been identified to have persistent oral HPV-16 DNA infection. Following discussion with our ethics team we have approached these three participants and offered them consultation with an Ear, Nose, and Throat (ENT) surgeon. A 63-year-old caucasian male was assessed as part of this consultation process. He had consistently been HPV-16 DNA positive for a period of 36 months, with a steadily rising HPV-16 viral load in his salivary oral rinse samples (Figure 1A). He was invited to attend the ENT clinic for assessment and discussion.

He is an ex-smoker, having quit 15 years ago, with a 45 pack year history of smoking. He drinks two standard drinks (2.5 units of alcohol) per day. He is heterosexual, and his social history includes multiple oral sex partners in the past (>5), followed by a long term monogamous relationship. Initial clinical examination of the oropharynx including palpation and white light revealed no significant abnormalities. Both tonsils were irregular due to mucous retention cysts and there was slight tonsillar asymmetry (Left < Right) but no evidence of any malignant lesions. Narrow band imaging (NBI) showed some mild vascular changes at the left glosso-tonsillar sulcus. There were no palpable lymph nodes in the neck. An MRI examination of the oropharynx and neck demonstrated no occult lesions of the tonsils or the base of tongue and no cervical lymphadenopathy.

He was offered continued surveillance, or a biopsy of the area of NBI change with bilateral tonsillectomy. The patient elected for bilateral tonsillectomy and biopsy of the base of tongue with NBI guidance under general anesthetic and informed consent was obtained. The surgical specimens were sent for histology and tissue HPV-16 DNA testing. The patient was discharged from hospital the same day. He had a routine postoperative course with a sore throat for 1 week and recovered fully. An ultrasound scan of his neck was performed 2 months post-surgery which showed no cervical lymphadenopathy. He is currently under routine oncological surveillance. The patient has a very high likelihood of cure with minimal morbidity from single modality treatment.

## Clinical Specimens' Collection and Processing

Salivary oral rinse samples of this individual were collected at baseline, 6, 12, 36 month, and 2 weeks after his bilateral tonsillectomy using previously published method (8–10). Briefly, participants were asked to swish and gargle for 1–2 min with 2 × 10 mL volumes of 0.9% saline, prior to expectorating the rinse sample into a 50 mL falcon tube. Tissue biopsies from the tonsil and base of tongue were obtained after surgical resection. All samples were immediately frozen on dry ice upon collection and transported back to the laboratory for subsequent processing.

## HPV-16 DNA QPCR Analysis

Total DNA was extracted from salivary oral rinse and tonsillar tissue samples using the QIAmp DNA Mini Kit (Qiagen, Germantown, MD, USA) as per manufacturer's protocol. For detection of HPV-16 genotyping, the qPCR assay targeting the opening reading frame (ORF) region of HPV16 E6/7 was carried out with the QuantStudio™ 7 Flex Real-Time PCR System (Applied Biosystems, Foster City, CA, USA) as described previously (11, 12). For quantification of HPV-16 DNA viral copies in salivary oral rinse and tissue samples, a standard calibration curve was generated using qPCR by plotting threshold cycle (Ct values) against the logarithm of the copy number of 8-fold serially diluted (1 × 10^1^-1 × 10^8^ copies) of pHPV-16 plasmid DNA [American Type Culture Collection (ATCC)® 45113™].

## Immunohistochemistry

H&E (Haemotoxylin and Eosin stains) staining on formalin-fixed paraffin-embedded (FFPE) slide was performed to investigate the cellular and tissue structure/morphology. HPV status was evaluated using CINtec® p16INK4a Histology Kit (E6H4 clone) (Roche MTM Laboratories, Heidelberg, Germany) according to manufacturer's instructions. p16INK4a was considered positive by two independent pathologists when there was a strong, diffuse nuclear and cytoplasmic staining pattern in the majority (>70%) of tumor cells.

## Salivary HPV-16 DNA as a Biomarker-Based Tool for HPV-Driven OPC Screening

Salivary oral rinse samples from this individual had been collected at baseline, 6, 12, and 36 month follow-up as well as 2 weeks after his bilateral tonsillectomy. HPV-16 DNA genotyping and viral loads in all samples were analyzed using an in-house developed qPCR assay. HPV-16 DNA viral load in saliva increased exponentially across the 36 month follow-up period (from 3.43 to 1,281.69 copies/50 ng) and subsequently declined to undetectable levels post-tonsillectomy (Figure 1A).

## Histologically Confirmed Diagnosis of an Occult P16INK4A Positive OPC

This individual was diagnosed as having a 2 mm squamous cell carcinoma (T1N0M0) in the left tonsil (Figure 1B) using Haemotoxylin and Eosin (H&E) staining. He had only foci of stromal invasion with a depth of <1 mm. The remainder of the left tonsil showed follicular lymphoid hyperplasia. Further, HPV-16 DNA was only positive in left tonsillar tissue (Figure 1C). Immunohistochemistry (IHC) staining for p16INK4a demonstrated diffuse and strong staining in more than 70% of tumor cells (Figure 1D). However, the non-affected remainder of the left tonsil as well as the right tonsil were negative for p16INK4a with usual mosaic pattern of staining. The excision margins of the left tonsillar malignancy were widely clear. No atypia or malignancy could be identified in the right tonsil and bilateral tongue base specimens all of which were negative for HPV-16 DNA.

## Discussion

Long-term persistence of HPV-16 infection is likely to be a prerequisite for the development of malignancy (13, 14). Women with persistent HPV-16 infection in the cervix for <1 year have a 40% risk of developing cervical intraepithelial neoplasia grade 2 or more within 3 years (13). Indeed, the natural history of HPV in the oropharynx from initial infection to carcinogenesis is not known with many questions remaining unanswered. Several studies have evaluated the prevalence of HPV-16 DNA in saliva (15–18) without clinical assessment of positive individuals. Studies aimed at clinical assessment of those with persistence for premalignancy or microscopic carcinoma have failed to detect significant abnormalities (19). This has led to the assertion that screening for early occult or premalignant oropharyngeal lesions is not feasible. Here, we report the first ever histologically confirmed diagnosis of an asymptomatic occult OPC (T1N0M0) discovered by a theoretical screening test through the serial measurements of HPV-16 DNA in salivary oral rinse samples.

The impact of the pattern of salivary HPV persistence including changes in the absolute HPV viral DNA copies over time has never been investigated. The pattern of salivary HPV-DNA detection in this case demonstrates an exponential upward trend with the titer at first sample being 3.43 copies per 50 ng and the final titer before surgery of 1281.7 viral copies per 50 ng. This may represent progression of the lesion with subsequent shedding of increasing levels of HPV-16 DNA into the saliva. In future cases the presence of this pattern should be evaluated, as it may provide a critical marker for the progression of disease and hence a signal for intervention; indeed the pattern of viral copies in serial measurement may have more importance than the persistence itself.

This case also has important implications with regards to the natural history of the disease. The left tonsil was strongly positive for HPV 16 DNA outside the region of malignancy and as anticipated was p16INK4a positive only within carcinoma. This implies that the malignancy is likely to have developed in a wider field of HPV infection with only a component undergoing malignant change. The existence of a precursor lesion to OPC has long been doubted and is cited as one of the obstacles to OPC screening (16). This case demonstrates that very early lesions can be found in asymptomatic individual, and that they can potentially be eradicated with minimal morbidity.

The quest for a sensitive and specific screening test for HPV-driven OPC is of great importance. The uptake of HPV immunization in developed countries is variable and the developing world remains largely unimmunised. As sexual habits change in the developing world (20, 21) there is likely to be the same rapid expansion in this disease that we have witnessed in the United States and Europe and global burden will continue to rise. As the first singular case, this report does not act as direct evidence of the value of screening in a general population, however, it demonstrates a possible salivary screening test and pathway for the detection of microscopic OPC. It demonstrates that a screened individual can receive significantly less morbid treatment than would be required for the standard presentation at a more advanced stage. This report and previous studies (8, 11, 12, 22), support the value of a salivary oral rinse test as a potential screening tool. Unlike previously published work, our study is the first to demonstrate that continuous monitoring of HPV-16 DNA in salivary oral rinse samples can detect occult OPC.

## Data Availability Statement

All datasets generated for this study are included in the article.

## Ethics Statement

This study was approved by institutional ethics committees from the University of Queensland (UQ) [HREC No: 2014000679 and 2014000862]; Queensland University of Technology [HREC No: 1400000617 and 1400000641]; and the Royal Brisbane and Women's Hospital (RBWH) [HREC/16/QRBW/447]. Written informed consent was obtained from this participant for publication of this case report.

## Author Contributions

All authors have read and agree to the published version of the manuscript. KT and CP: conceptualization. All authors: methodology, validation, formal analysis, data curation, investigation, and writing—review and editing. KT, SV, and CP: writing—original draft preparation. CP: funding acquisition.

### Conflict of Interest

The authors declare that the research was conducted in the absence of any commercial or financial relationships that could be construed as a potential conflict of interest.
